# *Cyperus esculentus* L. Tubers (Tiger Nuts) Protect Epithelial Barrier Function in Caco-2 Cells Infected by *Salmonella* Enteritidis and Promote *Lactobacillus plantarum* Growth

**DOI:** 10.3390/nu13010071

**Published:** 2020-12-28

**Authors:** David Moral-Anter, Joan Campo-Sabariz, Ruth Ferrer, Raquel Martín-Venegas

**Affiliations:** 1 Secció de Fisiologia, Departament de Bioquímica i Fisiologia, Facultat de Farmàcia i Ciències de l’Alimentació, Universitat de Barcelona, 08028 Barcelona, Spain; davidmoral@ub.edu (D.M.-A.); joan_campo@ub.edu (J.C.-S.); rutferrer@ub.edu (R.F.); 2 Institut de Recerca en Nutrició i Seguretat Alimentària (INSA-UB), Universitat de Barcelona, 08291 Santa Coloma de Gramenet, Spain

**Keywords:** paracellular permeability, ZO-1, occludin, TNFα, H_2_O_2_, bacterial agglutination

## Abstract

*Cyperus esculentus* L. tubers (tiger nuts) contain different compounds with several intestinal health-promoting properties. Here, we studied the capacity of tiger nuts from Valencia, Spain, to prevent epithelial barrier function disruption induced by *Salmonella* enteritidis in Caco-2 cell cultures. Paracellular permeability was assessed by transepithelial electrical resistance (TER) and tight junction protein immunolocalization. Moreover, the effect of tiger nuts on *S.* enteritidis agglutination, oxidative stress, and *Lactobacillus plantarum* growth was tested. Compared to controls, tiger nuts partially restored TER in *S.* enteritidis-infected cultures, an effect confirmed by immunolocalization of tight junction proteins ZO-1 and occludin. The results also revealed that this protective effect may be associated with the capacity to agglutinate the pathogen, restore TER in TNFα-stimulated cultures, and reduce reactive oxygen species in H_2_O_2_-stimulated cultures. Moreover, they favor *L. plantarum* growth. In conclusion, this study demonstrates that the tiger nut protects epithelial barrier function by reducing bacterial invasion, along with counteracting TNFα and H_2_O_2_ effects, thus giving an additional value to this tuber as a potential functional food.

## 1. Introduction

*Cyperus esculentus* L. (*Cyperus esculentus* L. var. *sativus* Boeck.), of the Cyperaceae family, is a perennial herb with rhizomes ending in hard tubers. These tubers (tiger nuts) are rounded or oval, thickened, with brownish folded skin, and full of reserve substances. Currently, the herb is mainly cultivated in Southern Europe (region of Valencia, Spain), North and South America, Africa, Australia, and China. The consumption of tiger nuts is very popular because these tubers can be consumed raw [1,2] or processed to obtain a milky beverage (commonly known in the Catalan as orxata), flour, and oil [3]. Despite its extended use in certain regions of the world, there is little available information about potentially beneficial uses. In this regard, it has been reported that tiger nut milk prevents liver injury in rats [4]. Moreover, the administration of an aqueous extract to rats for 30 days lowered blood glucose levels, suggesting a possible role in diabetes treatment [5]. In fact, Valencian tiger nut has been described to contain a wide variety of compounds (Table 1) [6,7,8], among others, that could help maintain or improve intestinal barrier function.

There is clear evidence that the alteration of intestinal barrier function plays a key role in the pathogenesis of different intestinal diseases. In particular, the infection produced by different strains of the Gram-negative bacteria *Salmonella* spp. is a major cause of foodborne illness throughout the world. Human salmonellosis, induced by *Salmonella* enteritidis, is associated with the consumption of contaminated foodstuffs, mainly poultry and eggs [9]. In most cases, the symptoms of salmonellosis are relatively mild, and patients recover without specific treatment. However, in some cases, particularly in young children and in the elderly, dehydration caused by the disease can be serious and life-threatening.

After ingestion, *Salmonella* possesses mannose-specific lectins in type-1 fimbriae that adhere to glycoproteins of the intestinal epithelium [10]. Then, the bacterium delivers virulence proteins encoded by *Salmonella* pathogenicity island (SPI) into the host cell cytoplasm [11,12]. The SPI1 and SPI2 secretion systems induce the secretion of inflammatory mediators such as tumor necrosis factor alpha (TNFα), interleukin 1β (IL-1β), and IL-18, which alter the epithelial barrier function and trigger the inflammatory response [13]. Moreover, previous results obtained by our group indicate that not only TNFα, but also oxidative stress may be an important factor in the capacity of *S.* enteritidis to disrupt epithelial barrier function [14,15]. 

In light of the above, the aim of this study was to investigate the potentially beneficial role of Valencian tiger nuts (*Cyperus esculentus* L. var. *sativus* Boeck tubers) in preventing epithelial barrier function disruption induced by *S.* enteritidis in an in vitro model of intestinal Caco-2 cells in culture; whether through its antimicrobial, anti-inflammatory, and/or antioxidant properties. Moreover, the capacity of tiger nuts to stimulate the growth of the proven probiotic *Lactobacillus plantarum* was also tested.

## 2. Materials and Methods 

### 2.1. Material

Dulbecco’s modified Eagle’s medium (DMEM), trypsin, penicillin, streptomycin, and ProLong™ Gold Antifade Mountant were supplied by Life Technologies (Carlsbad, CA, USA). Nonessential amino acids, sterile phosphate-buffered saline (Dulbecco’s phosphate-buffered saline (DPBS)), dimethyl sulfoxide (DMSO), bovine serum albumin (BSA), H_2_O_2_, and other chemicals were purchased from Sigma-Aldrich (St. Louis, MO, USA). Fetal bovine serum (FBS) was purchased from GE Healthcare Life Sciences (Issaquah, WA, USA). TNFα was supplied by Enzo Life Sciences (Farmingdale, NY, USA). Tryptic soy agar (TSA) and De Man, Rogosa, and Sharpe (MRS) agar were purchased from Thermo Fisher Scientific Oxoid (Hampshire, UK). Tissue culture supplies, including Transwells^®^, were obtained from Costar (Cambridge, MA, USA).

The study was carried out with tubers supplied by Regulatory Council D.O. Tiger Nut of Valencia (*Cyperus esculentus* L. var. *sativus* Boeck, Protected Designation of Origin). Whole tubers were crushed with a grinder until a flour was obtained (referred to in this article as tiger nut). Then, the flour was homogenized (Polytron, Kinematica AG, Luzern, Swiss) in DMSO (50 mg tiger nut/mL DMSO). In all experiments, the flour was further diluted to 2.5 mg/mL with DMEM. 

### 2.2. Caco-2 Cell Culture

Caco-2 cells were purchased from ECACC (Salisbury, UK). The cells were routinely grown in plastic flasks and cultured at a density of 10^4^ cells/cm^2^, as previously described [16]. Cells were subcultured on polycarbonate filters with a pore size of 0.4 µm (Transwells^®^, 12 mm diameter) for cell viability, paracellular permeability experiments, and tight junction protein immunolocalization; on 24 well clusters to determine intracellular reactive oxygen species (ROS), and on glass coverslips for scanning electron microscopy. Experiments were performed in DMEM when cells were differentiated [16].

Cells were pre-incubated over 24 h with 2.5 mg/mL tiger nut, and then, a sample of the culture media was taken to evaluate Caco-2 cell viability by measuring lactate dehydrogenase (LDH) activity, as previously described [14]. Then, the cultures were incubated with *S*. enteritidis (multiplicity of infection (MOI) 10, for 3 h), or TNFα (300 ng/mL, for 48 and 72 h), or H_2_O_2_ (350 µmol/L, for 3 h) [14,15]. In all experimental conditions, including control cultures, the final DMSO concentration was 5 %. This concentration of DMSO does not affect either cell viability or transepithelial electrical resistance (TER) values (data not shown).

### 2.3. Bacterial Growth

*S*. enteritidis (phage type 4, nalidixic acid-resistant strain) was provided by Dr. Ignacio Badiola from the Centre de Recerca en Sanitat Animal (CReSA, IRTA-UAB, Bellaterra, Spain) and prepared as previously described [14]. The effect of tiger nut on *S*. enteritidis growth was investigated in a bacterial suspension in DMEM with the same bacterial concentration and incubation time as in the culture experiments (Section 2.2). At the end of the incubation period, a sample of the suspension was serially diluted in DPBS and plated onto TSA at 37 °C for 24 h. Then, colony-forming units (CFUs) were counted.

*L. plantarum* (American Type Culture Collection, ATCC 8014) was provided by Dr. Ana Marqués (Facultat de Farmàcia i Ciències de l’Alimentació, Universitat de Barcelona) and prepared as previously described [15]. The effect of tiger nut on *L. plantarum* growth was investigated in a bacterial suspension in DMEM (6·10^6^ bacteria/mL). The suspension was incubated for 24 h at 37 °C, and at the end of the incubation period, a sample of the suspension was serially diluted in DPBS and plated onto MRS agar at 37 °C for 48 h. Then, CFUs were counted.

### 2.4. Paracellular Permeability and Confocal Microscopy

After incubation with *S*. enteritidis or TNFα, transepithelial electrical resistance (TER) was measured, as described elsewhere [16]. Moreover, at the end of the incubation period with *S*. enteritidis, occludin and *zonula occludens*-1 (ZO-1) immunolocalization were evaluated. Briefly, Caco-2 monolayers were fixed, permeabilized, and blocked, as previously described [14]. Cells were incubated overnight with primary antibodies at 4 °C. Mouse monoclonal anti-occludin (1:50 dilution; Life Technologies) and rabbit polyclonal anti-ZO-1 (1:50 dilution; Life Technologies) were used as primary antibodies. Monolayers were then incubated for 45 min with Alexa dye-conjugated secondary antibodies (1:300 dilution; Alexa Fluor 488 donkey anti-rabbit and Alexa Fluor 555 goat anti-mouse, Life Technologies). Finally, cells were incubated with Hoechst (5 µL/mL, 5 min; Life Technologies) to view the nucleus and mounted in ProLong™ Gold Antifade Mountant. The samples were examined with a confocal laser scanning microscope (TCS-SP5; Leica Lasertechnik, GmbH, Germany) at the Centres Científics i Tecnològics of the Universitat de Barcelona. Images were processed by ImageJ software (public domain, National Institutes of Health). The nuclei of *S*. enteritidis were counted at the microvillous level.

### 2.5. Scanning Electron Microscopy

After *S*. enteritidis incubation, Caco-2 cell cultures were prepared for scanning transmission electron microscopy, as previously described [14], and then processed and examined in a Zeiss DSM 940A (Oberkochen, Germany) microscope at the Centres Científics i Tecnològics of the Universitat de Barcelona.

### 2.6. Intracellular Reactive oxygen Species 

After H_2_O_2_ incubation, intracellular ROS generation was evaluated using a commercial intracellular ROS assay kit (OxiSelect™, Cell Biolabs Inc., Bionova, Barcelona), as previously described [14]. 

### 2.7. Statistical Analysis

Data were analyzed by one-way ANOVA followed, where needed, by Bonferroni’s post hoc tests, using IBM SPSS Statistics 22 (SPSS Inc. Chicago, IL, USA). *p* < 0.05 was considered to denote statistical significance. 

## 3. Results

### 3.1. Effect of Tiger Nut on Caco-2 Cell Viability and TER

Caco-2 cell incubation with 2.5 mg/mL tiger nut did not produce statistically significant differences in LDH activity in comparison to control cells (control: 100.0 ± 3.2 % and tiger nut: 102.3 ± 2.3 %, *p* > 0.05). In fact, Chukwuma et al. [5] indicated an absence of undesirable effects in rats, using a concentration between 100- and 400-fold higher than that used herein. Moreover, tiger nut did not affect TER values after 72 h of incubation in comparison to control cells (control: 2446 ± 28.4 Ω·cm^2^ and tiger nut: 2546 ± 49.0 Ω·cm^2^, *p* > 0.05). Thus, this concentration was used in subsequent experiments.

### 3.2. Effect of Tiger Nut on Paracellular Permeability in Caco-2 Cells Incubated with S. enteritidis

**Figure 1:**
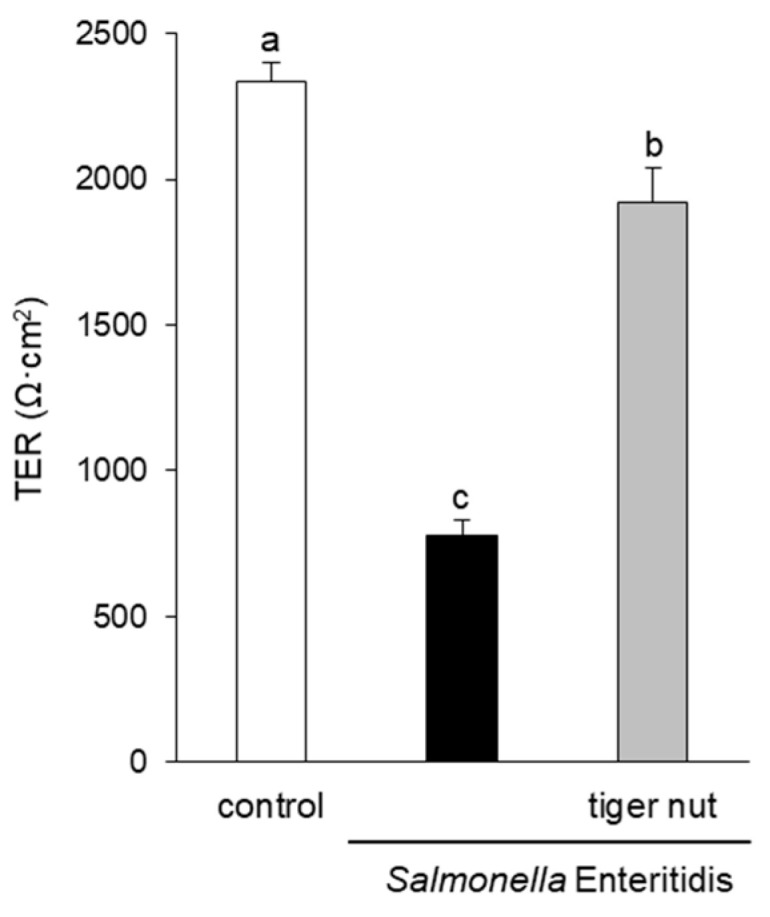
Effect of tiger nut on transepithelial electrical resistance (TER) in Caco-2 cells incubated with *S*. enteritidis. Cultures were incubated for 3 h in the absence (control) or presence of *S*. enteritidis (MOI 10) and tiger nut (2.5 mg/mL). Results are expressed as the mean ± SEM of n = 9 monolayers. Labeled bars without a common letter differ (*p* < 0.05).

**Figure 2:**
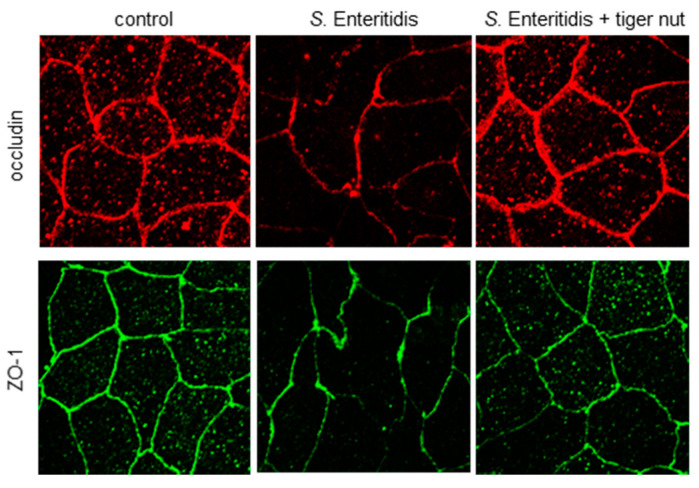
Effect of tiger nut on the immunolocalization of occludin and ZO-1 in Caco-2 cells incubated with *S*. enteritidis. Cultures were incubated for 3 h in the absence (control) or presence of *S*. enteritidis (MOI 10) and tiger nut (2.5 mg/mL). Representative results were reproduced in three separate experiments.

In *S*. enteritidis infected cells, the presence of tiger nut conferred a partial protection of TER, which was significantly decreased in infected cultures (Figure 1). Regarding the distribution of tight junction proteins (Figure 2), in control cultures, occludin and ZO-1 were visualized delineating the cellular borders. In cultures infected with *S*. enteritidis, the images revealed that both proteins showed a reduction in fluorescence and lost their outline. In infected cells, incubation with tiger nut showed a distribution and intensity of fluorescence, for both proteins, similar to that of the control cultures.

### 3.3. Effect of Tiger Nut on S. Enteritidis Adhesion to the Epithelium

**Figure 3:**
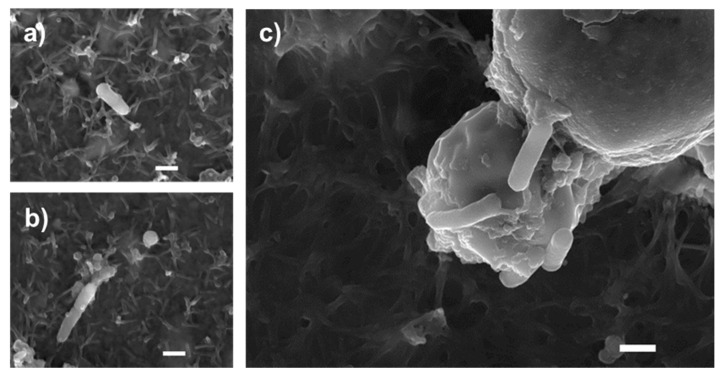
Bacterial agglutination capacity of tiger nut. SEM images of cultures incubated for 3 h (**a**,**b**) in the presence of *S*. enteritidis (MOI 10), and (**c**) in the presence *S*. enteritidis (MOI 10) in addition to tiger nut (2.5 mg/mL). Representative results were reproduced in three separate experiments. The white bar represents 1 µm.

**Figure 4:**
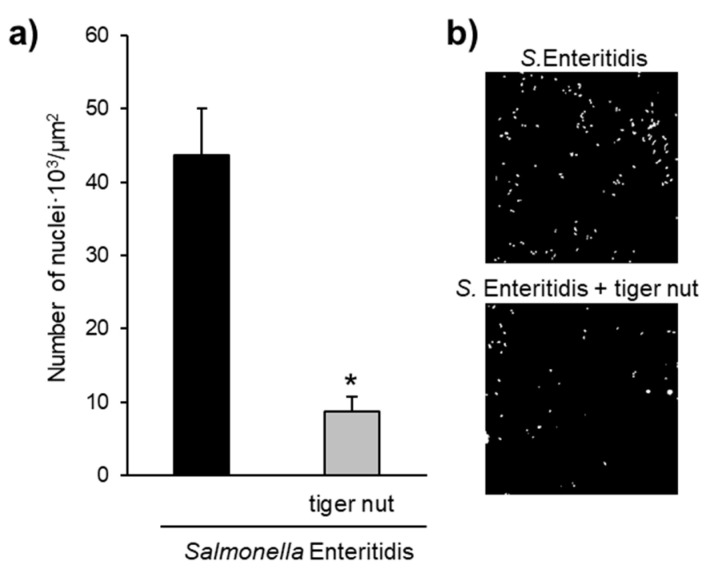
Effect of tiger nut on *S*. enteritidis counts at the microvillous level. (**a**) Number of nuclei · 10^3^/µm^2^ of culture surface in cultures incubated for 3 h in the presence of *S*. enteritidis and in the presence *S*. enteritidis (MOI 10) in addition to tiger nut (2.5 mg/mL). Nuclei were not detected in control cultures. Results are expressed as the mean ± SEM of *n* = 3 experiments. The asterisk denotes statistical differences (*p* < 0.05). (**b**) Confocal images of culture surface in cells incubated for 3 h in the presence of *S*. enteritidis (MOI 10) and in the presence of *S*. enteritidis (MOI 10) in addition to tiger nut (2.5 mg/mL). Micrographs correspond to a presentative image from three separate experiments. Results are expressed as the mean ± SEM of *n* = 3 experiments.

**Figure 5:**
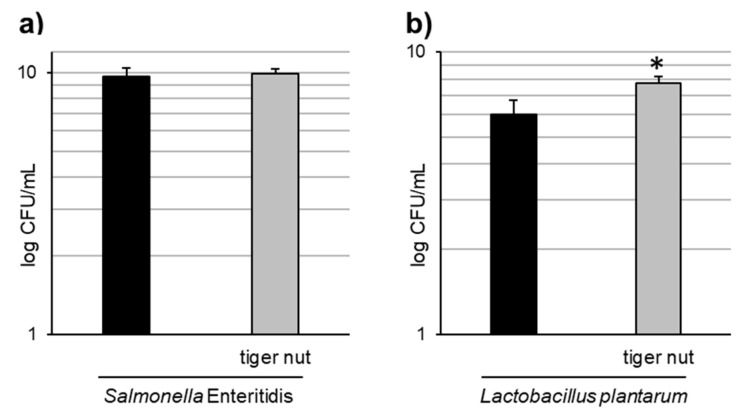
Effect of tiger nut on bacterial growth. (**a**) *S*. enteritidis growth evaluated in cultures incubated for 3 h in the absence or presence of tiger nut (2.5 mg/mL). Results are expressed as the mean ± SEM of *n* = 6 experiments. (**b**) *L. plantarum* growth evaluated in cultures incubated for 24 h in the absence or presence of tiger nut (2.5 mg/mL). Results are expressed as the mean ± SEM of n = 5 experiments. The asterisk denotes statistical differences (*p* < 0.05).

Images obtained by scanning electron microscopy revealed that *S.* enteritidis is preferably attached to the surface of tiger nut, thus presumably reducing bacteria adhered to the epithelium (Figure 3). In fact, *S*. enteritidis at the microvillous level is reduced by the addition of tiger nut (Figure 4). Moreover, the data also indicated that tiger nut did not affect the viability of *S.* enteritidis (Figure 5a).

### 3.4. Effect of tiger nut on TER and ROS production in Caco-2 cells incubated with TNFα and H_2_O_2_

**Figure 6:**
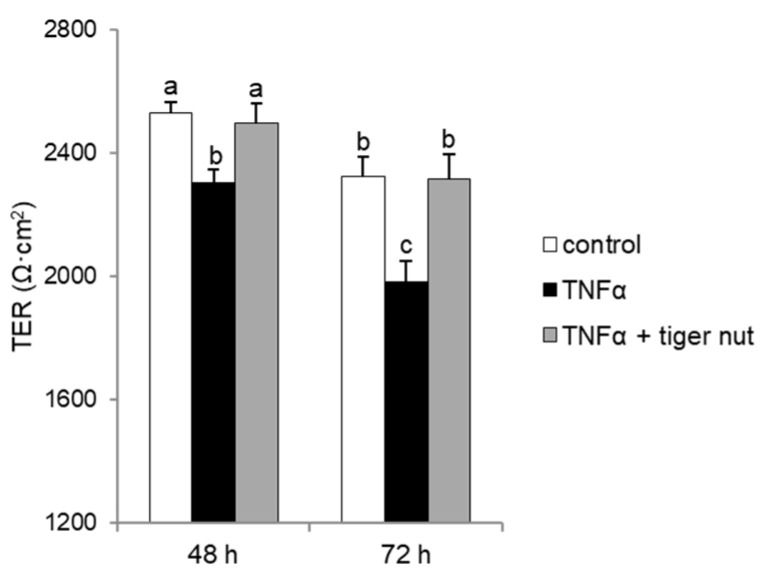
Effect of tiger nut on TER in Caco-2 cells incubated with TNFα. Cultures were incubated for 48 and 72 h in the absence (control) or presence of TNFα (300 ng/mL) and tiger nut (2.5 mg/mL). Results are expressed as the mean ± SEM of *n* = 12 monolayers. Labeled bars without a common letter differ (*p* < 0.05).

**Figure 7:**
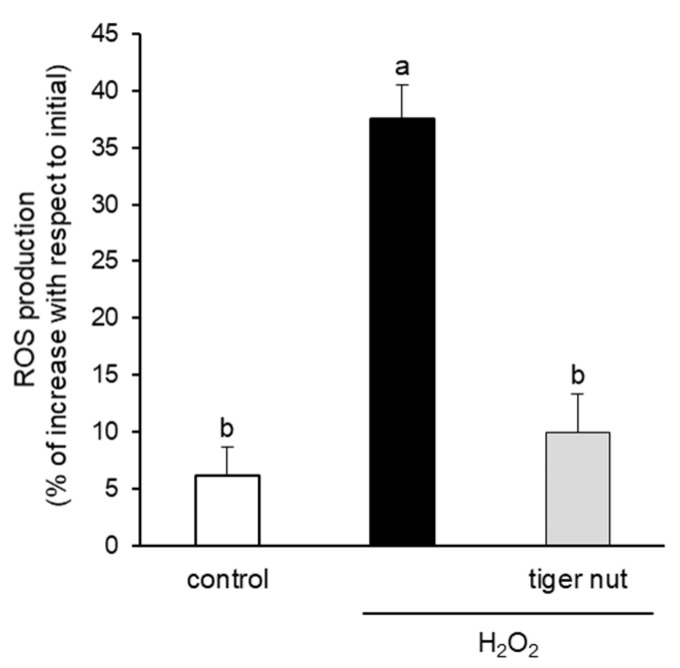
Effect of tiger nut on ROS production in Caco-2 cells incubated with H_2_O_2_. Cultures were incubated for 3 h in the absence (control) or presence of H_2_O_2_ (350 µmol/L) and tiger nut (2.5 mg/mL). Results are expressed as the mean ± SEM of *n* = 16 monolayers. Labeled bars without a common letter differ (*p* < 0.05).

With the objective to further investigating the mechanism underlying the protective role of tiger nut on *S.* enteritidis infection, independently of the capacity to agglutinate the bacterium, its effect was tested in cultures stimulated with TNFα and H_2_O_2_. As expected, the results showed that the stimulation with TNFα decreased TER values at 48 and 72 h (Figure 6). Interestingly, the incubation of TNFα-stimulated cultures with tiger nut showed similar values to the control cells at 48 and 72 h. Similarly, the ROS values of H_2_O_2_-incubated cells were fully restored in the presence of tiger nut, showing no statistically significant differences from control cells (Figure 7). 

### 3.5. Effect of Tiger Nut on L. Plantarum Growth

As tiger nut contains dietary fiber and other components with a possible prebiotic activity, tests were conducted to determine whether this tuber may have an effect on the growth of *L. plantarum*. The results indicated a statistically significant increase in the growth of this strain in the presence of tiger nut (Figure 5b), an effect that was also observed even at a 10-fold lower concentration of tiger nut (data not shown).

## 4. Discussion

One of the main goals of this study was to investigate the potential beneficial role of tiger nut in preventing epithelial barrier function disruption induced by *S.* enteritidis colonization. As expected, the infection resulted in an increase in the paracellular permeability of the intestinal epithelium, as well as a disorganization of occludin and ZO-1. The ability of this bacterium to disrupt epithelial barrier function has been widely described [14,15,17,18,19,20,21]. Interestingly, tiger nut provided a protective role against the consequences of this infection. 

*S.* enteritidis possesses mannose-specific lectins in type-1 fimbriae that are responsible for binding to the mannose of the glycoproteins on the surface of the host cell and thus invading the epithelium [22,23]. Therefore, one possible hypothesis to explain the protective effect of tiger nut on epithelial barrier function would be that certain carbohydrates present in this tuber, mainly starch and dietary fiber [1], could compete with epithelial binding sites and bind *S.* enteritidis, preventing (or decreasing) its invasive capacity. In this sense, tiger nut has a high content of dietary fiber [6]. Indeed, this effect has already been described for a β-galactomannan developed from the carob bean of the *Ceratonia siliqua* tree and the guar bean of *Cyamopsis tetragonoloba* [24,25]. The images obtained by scanning electron microscopy revealed the capacity of *S.* enteritidis to adhere to tiger nut, thus presumably reducing the adhesion of the bacterium to intestinal epithelial cells. Moreover, the density of *S*. enteritidis nuclei decreases with the addition of tiger nut. 

Another hypothesis to explain this protective effect is that tiger nuts may have an antibiotic effect, as it has been suggested to have against other bacterial species [3]. However, the possible antibiotic effect of tiger nut at the concentration tested is ruled out here. In fact, Adeniyi et al. [26] reported that the antimicrobial activity of tiger nut is found at a concentration much higher than that used here (100 mg/mL vs. 2.5 mg/mL) and that this activity is much lower than that of classical antibiotics.

The interaction of *S.* enteritidis with the intestinal epithelium, as well as its invasion, triggers diverse transduction signals at the epithelial and subepithelial compartments, which induce the activation of immune cells and therefore the onset of the inflammatory response [11]. The secretion of proinflammatory cytokines disrupts epithelial barrier function, which, in turn, contributes to water loss and bacterial translocation, perpetuating the inflammatory process and initiating systemic invasion [27]. In this sense, TNFα is a proinflammatory cytokine known to induce an inflammatory response in intestinal epithelial cells and to impair the tight junction structure [28,29]. Since *Salmonella* infection induces an increase in TNFα production, the effect of infection on paracellular permeability can be, in part, attributed to this cytokine [15,24]. Therefore, the data obtained here in cultures stimulated with TNFα, revealed another pathway to explain the protective role of tiger nut against *S*. enteritidis, independently of its capacity to avoid infection by agglutinating the bacterium. Tiger nut has a moderately high content of tocopherols, the proportion of α-tocopherol being higher than the proportion of β- and γ-tocopherol. The main function of vitamin E is its natural antioxidant capacity, α-tocopherol having the greatest potency [7,8]. In this sense, it has been described that vitamin E administration reduces TNFα production in rats treated with a pesticide [30], as well as increasing occludin expression and decreasing NFκB expression in the intestine of rats exposed to a high altitude hypoxia environment [31]. Therefore, these results suggest that tiger nut could be effective in intestinal inflammatory processes. 

Epithelial barrier function is also regulated by oxidative stress. In this case, oxidative stress induces phosphorylation of tight junction proteins, causing the dissociation of occludin and ZO-1 and, as a consequence, their dissociation from cytoskeletal proteins, altering epithelial barrier function [16,32]. In a previous work, we observed that the incubation of Caco-2 cells with *S.* enteritidis increases intracellular ROS and secondary oxidation products [14]. In addition, Banan et al. [33] observed that in Caco-2 cells, H_2_O_2_ has the ability to reduce the amount of occludin associated with the cytoskeleton completely and with ZO-1 partially, as well as to disorganize the actin of the cytoskeleton. Therefore, the capacity of tiger nut to counteract the effect of H_2_O_2_ may suggest that the antioxidant capacity, mainly ascribed to its polyphenol and α-tocopherol contents [8], could also be responsible for the recovery of epithelial barrier function induced by oxidative stress. Along these lines, there have been descriptions of an increase in reduced glutathione in the liver, kidney, and heart among rats fed a diet based on tiger nut oil [34].

In this study, it can be observed that tiger nut has a prebiotic effect on *L. plantarum*, and thus, it could stimulate the growth of gut microbiota, which could favor the removal of pathogens. In this vein, *L. plantarum* has been described as able to reduce *Salmonella* typhimurium infection in chicks as it has the ability to restore intestinal permeability by modifying the expression of tight junction proteins and by decreasing pathogen colonization [35]. Indeed, it has been shown that this bacterium could have the ability to stabilize tight junctions through the inhibition of extracellular signal-regulated kinases (ERK) [36]. It has previously been suggested that the starch content of a tiger nut liquid co-product provides prebiotic properties for colon bacteria [37]. Sánchez-Zapata et al. [38] had already shown that this co-product favored the growth of probiotic strains of *Lactobacillus acidophilus* and *Bifidobacterium animalis*. In the same vein, almonds, with a similar composition as tiger nut, promoted the growth of *L. acidophilus* and *Bifidobacterium brief* [39] and increased the production of butyric acid [40], a short-chain fatty acid closely associated with gut health.

In conclusion, the protection offered by the tiger nut against *S*. enteritidis infection in Caco-2 cell cultures could be explained by its ability to reduce bacterial invasion, as well as by its ability to counteract the effects of TNFα and H_2_O_2_. Furthermore, the results indicate that tiger nuts may have prebiotic properties. These properties highlight the value of this food, which represents a response to society’s demand for such potentially functional foods. Nevertheless, since changes in food matrix structures occur during digestion, further studies are needed to evaluate the bioaccessibility of the different compounds in the in vivo situation.

## Figures and Tables

**Table 1:** Composition of Valencian tiger nut.

	**g/100 g ^1^**	**Reference**
Water	8.83 ± 0.05	[6]
Protein	4.95 ± 0.07	[6]
Fat ^2^	25.07 ± 0.02	[6]
Ash	2.05 ± 0.04	[6]
Total dietary fiber ^3^	15.85 ± 0.03	[6]
DC^4^	43.25 ± 0.03	[6]
	**mg/100 g ^1^**	**Reference**
β-sitosterol	49 ± 3	[7]
ST + CA ^5^	11 ± 3	[7]
α-tocopherol	2.2 ± 0.3	[7]
β + γ- tocopherol	1.3 ± 0.5	[7]
	**µg/g oil ^6^**	**Reference**
Total polyphenols	13.2–24.6	[8]

^1^ Mean ± standard deviation. ^2^ Major fatty acids’ content (expressed as a percentage of the total fatty acid methyl esters): oleic acid (66.98), palmitic acid (13.87), linoleic acid (11.16), and stearic acid (5.98). ^3^ Soluble dietary fiber: 2.10 ± 0.03; insoluble dietary fiber: 13.74 ± 0.03. ^4^ Digestible carbohydrates calculated by the difference. ^5^ Stigmasterol (ST) + campesterol (CA). ^6^ Mean range depending on the extraction method.

## Data Availability

The data not shown presented in the study are available on request to the corresponding author.

## References

[1] Sánchez-Zapata E., Fernández-López J., Pérez-Álvarez J.A. (2012). Tiger nut (*Cyperus esculentus*) commercialization: Health aspects, composition, properties, and food applications. Compr. Rev. Food Sci. F.

[2] Ezeh O., Gordon M.H., Niranjan K. (2014). Tiger nut oil (*Cyperus esculentus* L.): A review of its composition and physico-chemical properties. Eur. J. Lipid Sci. Tech..

[3] Maduka N., Ire F.S. (2018). Tigernut plant and useful application of tigernut tubers (*Cyperus esculentus*)—A review. Curr. J. Appl. Sci. Technol..

[4] Onuoha N.O., Ogbusua N.O., Okorie A.N., Ejike C.E.C.C. (2017). Tigernut (*Cyperus esculentus* L.) “milk” as a potent “nutri-drink” for the prevention of acetaminophen-induced hepatotoxicity in a murine model. J. Intercult. Ethnopharmacol..

[5] Chukwuma E.R., Obioma N., Cristopher O.I. (2010). The phytochemical composition and some biochemical effects of Nigerian tigernut (*Cyperus esculentus* L.) tuber. Pak. J. Nutr..

[6] Martín-Esparza M.E., Raigón M.D., Raga A., Albors A. (2018). High fibre tiger nut pasta and xanthan gum: Cooking quality, microstructure, physico-chemical properties and consumer acceptance. Food Sci. Biotechnol..

[7] Delgado-Zamarreño M.M., Fernández-Prieto C., Bustamante-Rangel M., Pérez-Martín L. (2016). Determination of tocopherols and sitosterols in seeds and nuts by QuEChERS-liquid chromatography. Food Chem..

[8] Ezeh O., Niranjan K., Gordon M.H. (2016). Effect of Enzyme Pre-treatments on Bioactive Compounds in Extracted Tiger Nut Oil and Sugars in Residual Meals. J. Am. Oil Chem. Soc..

[9] European Food Safety Authority (2018). The European Union summary report on trends and sources of zoonoses, zoonotic agents and food-borne outbreaks in 2017. EFSA J..

[10] Spring P., Wenk C., Dawson K.A., Newman K.E. (2000). The effects of dietary mannan oligosaccharides on cecal parameters and the concentrations of enteric bacteria in the cecae of *Salmonella*-challenged broiler chicks. Poult. Sci..

[11] Wallis T.S., Galyov E.E. (2000). Molecular basis of *Salmonella*-induced enteritis. Mol. Microbiol..

[12] Sansonetti P. (2002). Host-pathogen interactions: The seduction of molecular cross talk. Gut.

[13] Reis R.S., Horn F. (2010). Enteropathogenic *Escherichia coli, Samonella, Shigella* and *Yersinia*: Cellular aspects of host-bacteria interactions in enteric diseases. Gut Pathog..

[14] Brufau M.T., Campo-Sabariz J., Bou R., Carné S., Brufau J., Vilà B., Marqués A.M., Guardiola F., Ferrer R., Martín-Venegas R. (2016). Salmosan, a β-Galactomannan-rich product, protects epithelial barrier function in Caco-2 Cells Infected by *Salmonella enterica* serovar Enteritidis. J. Nutr..

[15] Brufau M.T., Campo-Sabariz J., Carné S., Ferrer R., Martín-Venegas R. (2017). Salmosan, a β-galactomannan-rich product, in combination with *Lactobacillus plantarum* contributes to restore intestinal epithelial barrier function by modulation of cytokine production. J. Nutr. Biochem..

[16] Martín-Venegas R., Brufau M.T., Guerrero-Zamora A.M., Mercier Y., Geraert P.A., Ferrer R. (2013). The methionine precursor DL-2-hydroxy-(4-methylthio)butanoic acid protects intestinal epithelial barrier function. Food Chem..

[17] Solano C., Sesma B., Alvarez M., Urdaneta E., Garcia-Ros D., Calvo A., Gamazo C. (2001). Virulent strains of *Salmonella* enteritidis disrupt the epithelial barrier of Caco-2 and HEp-2 cells. Arch. Microbiol..

[18] Tafazoli F., Magnusson K.E., Zheng L. (2003). Disruption of epithelial barrier integrity by *Salmonella enterica* serovar typhimurium requires geranylgeranylated proteins. Infect. Immun..

[19] Bertelsen L.S., Paesold G., Marcus S.L., Finlay B.B., Eckmann L., Barrett K.E. (2004). Modulation of chloride secretory responses and barrier function of intestinal epithelial cells by the Salmonella effector protein SigD. Am. J. Physiol. Cell Physiol..

[20] Köhler H., Sakaguchi T., Hurley B.P., Kase B.A., Reinecker H.C., McCormick B.A. (2007). *Salmonella enterica* serovar Typhimurium regulates intercellular junction proteins and facilitates transepithelial neutrophil and bacterial passage. Am. J. Physiol. Gastrointest. Liver Physiol..

[21] Yu Q., Zhu L., Wang Z., Li P., Yang Q. (2012). *Lactobacillus delbrueckii* ssp. *lactis* R4 prevents *Salmonella* typhimurium SL1344-induced damage to tight junctions and adherens junctions. J. Microbiol..

[22] Althouse C., Patterson S., Fedorka-Cray P., Isaacson R.E. (2003). Type 1 fimbriae of *Salmonella enterica* serovar Typhimurium bind to enterocytes and contribute to colonization of swine in vivo. Infect. Immun..

[23] Sharon N. (2006). Carbohydrates as future anti-adhesion drugs for infectious diseases. Biochim. Biophys. Acta.

[24] Badia R., Brufau M.T., Guerrero-Zamora A.M., Lizardo R., Dobrescu I., Martín-Venegas R., Ferrer R., Salmon H., Martínez P., Brufau J. (2012). β-Galactomannan and *Saccharomyces cerevisiae* var. *boulardii* modulate the immune response against *Salmonella enterica* serovar Typhimurium in porcine intestinal epithelial and dendritic cells. Clin. Vaccine Immunol..

[25] Martín-Venegas R., Brufau M.T., Ferrer R., Muñoz-Torrero D., Vázquez-Carrera M., Estelrich J. (2014). Loss of intestinal epithelial barrier function in *Salmonella* Enteritidis infection. Recent Advances in Pharmaceutical Sciences IV.

[26] Adeniyi T.A., Adeonipekun P.A., Omotayo E.A. (2014). Investigating the phytochemicals and antimicrobial properties of three sedge (*Cyperaceae*) species. Not. Sci. Biol..

[27] Marchiando A.M., Graham W.V., Turner J.R. (2010). Epithelial barriers in homeostasis and disease. Annu. Rev. Pathol..

[28] Al-Sadi R., Boivin M., Ma T. (2009). Mechanism of cytokine modulation of epithelial tight junction barrier. Front. Biosci..

[29] Suzuki T. (2013). Regulation of intestinal epithelial permeability by tight junctions. Cell Mol. Life Sci..

[30] Sun Y., Zhang J., Song W., Shan A. (2018). Vitamin E alleviates phoxim-induced toxic effects on intestinal oxidative stress, barrier function, and morphological changes in rats. Environ. Sci. Pollut. Res. Int..

[31] Xu C., Sun R., Qiao X., Xu C., Shang X., Niu W., Chao Y. (2014). Effect of vitamin e supplementation on intestinal barrier function in rats exposed to high altitude hypoxia environment. Korean J. Physiol. Pharmacol..

[32] Rao R.K., Basuroy S., Rao V.U., Karnaky K.J., Gupta A. (2002). Tyrosine phosphorylation and dissociation of occludin-ZO-1 and E-cadherin-beta-catenin complexes from the cytoskeleton by oxidative stress. Biochem. J..

[33] Banan A., Fields J.Z., Zhang Y., Keshavarzian A. (2001). Phospholipase C-γ inhibition prevents EGF protection of intestinal cytoskeleton and barrier against oxidants. Am. J. Physiol. Gastrointest. Liver Physiol..

[34] Ibitoye O.B., Aliyu N.O., Ajiboye T.O. (2018). Tiger nut oil-based diet improves the lipid profile and antioxidant status of male Wistar rats. J. Food Biochem..

[35] Wang L., Li L., Lv Y., Chen Q., Feng J., Zhao X. (2018). *Lactobacillus plantarum* Restores Intestinal Permeability Disrupted by *Salmonella* Infection in Newly-hatched Chicks. Sci. Rep..

[36] Ko J.S., Yang H.R., Chang J.Y., Seo J.K. (2007). *Lactobacillus plantarum* inhibits epithelial barrier dysfunction and interleukin-8 secretion induced by tumor necrosis factor-alpha. World J. Gastroenterol..

[37] Alegría-Torán A., Farré-Rovira R., Fundación Valenciana de Estudios Avanzados (2003). Horchata y salud: Aspectos nutricionales y dietéticos. Jornada Chufa y Horchata: Tradición y Salud.

[38] Sánchez-Zapata E., Fuentes-Zaragoza E., Fernández-López J., Pintado M.M., Gomes A.M., Pérez-Álvarez J.A. Prebiotic properties of tiger nut (*Cyperus esculentus)* milk liquid co-products. Proceedings of the EFFoST Annual Meeting.

[39] Liu Z., Wang W., Huang G., Zhang W., Ni L. (2016). In vitro and in vivo evaluation of the prebiotic effect of raw and roasted almonds (*Prunus amygdalus*). J. Sci. Food. Agric..

[40] Mandalari G., Nueno-Palop C., Bisignano G., Wickham M.S., Narbad A. (2008). Potential prebiotic properties of almond (*Amygdalus communis* L.) seeds. Appl. Environ. Microbiol..

